# Topological Structure of the Space of Phenotypes: The Case of RNA Neutral Networks

**DOI:** 10.1371/journal.pone.0026324

**Published:** 2011-10-18

**Authors:** Jacobo Aguirre, Javier M. Buldú, Michael Stich, Susanna C. Manrubia

**Affiliations:** 1 Centro de Astrobiologa, CSIC-INTA, Madrid, Spain; 2 Complex Systems Group, Universidad Rey Juan Carlos, Madrid, Spain; 3 Laboratory of Biological Networks, Centre for Biomedical Technology, UPM-Campus de Montegancedo, Madrid, Spain; University of Zaragoza, Spain

## Abstract

The evolution and adaptation of molecular populations is constrained by the diversity accessible through mutational processes. RNA is a paradigmatic example of biopolymer where genotype (sequence) and phenotype (approximated by the secondary structure fold) are identified in a single molecule. The extreme redundancy of the genotype-phenotype map leads to large ensembles of RNA sequences that fold into the same secondary structure and can be connected through single-point mutations. These ensembles define neutral networks of phenotypes in sequence space. Here we analyze the topological properties of neutral networks formed by 12-nucleotides RNA sequences, obtained through the exhaustive folding of sequence space. A total of 4^12^ sequences fragments into 645 subnetworks that correspond to 57 different secondary structures. The topological analysis reveals that each subnetwork is far from being random: it has a degree distribution with a well-defined average and a small dispersion, a high clustering coefficient, and an average shortest path between nodes close to its minimum possible value, i.e. the Hamming distance between sequences. RNA neutral networks are assortative due to the correlation in the composition of neighboring sequences, a feature that together with the symmetries inherent to the folding process explains the existence of communities. Several topological relationships can be analytically derived attending to structural restrictions and generic properties of the folding process. The average degree of these phenotypic networks grows logarithmically with their size, such that abundant phenotypes have the additional advantage of being more robust to mutations. This property prevents fragmentation of neutral networks and thus enhances the navigability of sequence space. In summary, RNA neutral networks show unique topological properties, unknown to other networks previously described.

## Introduction

RNA is a well-suited model for studying evolution since genotype and phenotype are incorporated in a single molecular entity [1]. Built around a sugar-phosphate backbone, RNA consists of the 4 types of nucleotides ACGU and forms a unique sequence, representing genotype. Since the biochemical function of RNA is to a large extent given by its three-dimensional spatial conformation, the genotype-to-phenotype map of RNA can be split conceptually into a map from sequence to structure and a map from structure to function. Particularly for short sequences, the tertiary structure of an RNA molecule is very well approximated by the secondary structure fold. Therefore, RNA secondary structure represents one of the simplest possible realistic phenotypes [2], [3].

The mapping from sequence to secondary structure is many-to-one, i.e., there are many sequences that fold into the same structure. Assuming that all such sequences represent the same phenotype, they form a *neutral network* of genotypes. The number of different phenotypes gives the number of different neutral networks. The sequences that fold into the same secondary structure are the *nodes* of the neutral network. The *links* of the network connect sequences that are at a Hamming distance of one, i.e., that differ in only one nucleotide. Therefore, a neutral network may be connected – when all sequences are related to each other through single-point mutations – or disconnected. In the latter case, the neutral network is composed of a number of subnetworks. Examples can be found in [4].

Many structural aspects of the RNA sequence-structure map and of RNA neutral networks have been studied over the decades [2], [4]–[12], and have revealed a large part of the amazingly complex structure underlying the genotype-phenotype map. A rough upper bound to the number of different secondary structures *S_i_* retrieved by sequences of length *l*, and valid for sufficiently large sequences, was derived in [6]: 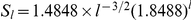. This implies that the average size of a neutral network grows as 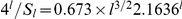, which is a huge number even for moderate values of *l*. This average value is however not representative of the actual distribution of neutral network sizes, which is a very broad function without a well-defined average and with a fat tail [6], [13]. The space of RNA sequences of length *l*, which is embedded in a regular lattice of dimension *l*, is dominated by a relatively small number of common structures which are extremely abundant and happen to be found as structural motifs of natural, functional RNA molecules [5], [14]. Neutral networks corresponding to common structures percolate the space of sequences [4], [8] and thus facilitate the exploration of a large number of alternative structures. This is possible since different neutral networks are deeply interwoven: all common structures can be reached within a few (mutational) steps starting from any random sequence [8]. In this contribution, we focus on the topology of RNA neutral networks and analyze local and global parameters describing their structure.

The application of complex networks theory to biological systems has given fruitful results about how the topology of the network is related to the dynamical processes occurring on it [15]–[17]. In protein-protein interaction networks, for example, nodes represent proteins that are connected through an undirected link if they bind to form a more complex component [18]. This kind of networks forms a giant connected component with small-world configuration (high clustering and short-path between nodes) [19], [20] and, in some cases, scale-free connectivity [20]–[22]. Networks with this structure are very robust against random failures and, at the same time, they are able to propagate any perturbation through the network within a few steps [23]. In the case of metabolic networks, nodes may represent metabolites, reactions or enzymes, and links between them have a given directionality. As in protein networks, the degree distribution shows scale-free connectivity [24], [25] and small-world structure [26]. In genetic regulatory networks, genes are the nodes of the network and transcription factors (activators or repressors) define directed links between nodes [27]. Again, despite being networks of different nature, the number of links leaving a certain node has a scale-free distribution [28], [29]. All of the biological networks listed in this paragraph result from constructive processes that preserve network functionality at all stages, modify the size of the networks through evolution, and optimize different biological traits. These processes are essential to determine the topological properties of the resulting networks. In this sense, their nature is different from RNA secondary structure neutral networks, whose topological characteristics are a consequence of the folding process. As will be shown, the local properties of neutral networks are constrained by the existence of four different nucleotides forming the RNA sequence and by the main structural motifs of the secondary structure (stacks and loops). An analysis of the restrictions they induce permits to obtain good analytical approximations to some of the topological features of neutral networks.

## Methods

### Sequence folding

We have folded *in silico* all different RNA sequences of length *l* = 12. As structure, we use the minimum free energy secondary structure, as predicted by routine fold( ) from the Program RNAfold of the Vienna RNA package [30], version 1.5, with the energy parameter set based on Ref. [31].

It must be noticed that RNAfold, as most folding programs, does not allow for pseudoknots or other kind of tertiary interactions. However, and in particular for the relatively short molecules considered here, secondary structures are a very good approximation of the tertiary structures since a major part of the folding energy corresponds to the secondary structure formation. No search for suboptimal structures was performed in this study.

RNA secondary structure folding consists in the formation of base pairs (through hydrogen bonding) between nucleotides of the same sequence (also called primary structure). The routine fold() is called with the default parameters, i.e., it allows Watson-Crick and G-U wobble base pairing (thus allowing in total 6 types of base pairs, G-C, C-G, A-U, U-A, G-U, U-G) and the temperature is set to 37°C. For a secondary structure the base pairs fulfill three conditions [3]: (a) An individual nucleotide participates in at most one base pair (no triplets or higher interactions). (b) Base pairs between nearest neighbors are excluded (actually, a hairpin loop must have at least size 3). (c) No pseudoknots: compared to any existing base pair, any other base pair either lies enclosed by the first one or lies completely outside. No special stabilizing energy contributions for tetraloops are assumed. Dangling end energies are assigned only to unpaired bases adjacent to stacks in free ends and multiloops. A base cannot participate simultaneously in two dangling ends. Single base pairs are allowed to form. Secondary structures are obtained in the standard bracket notation being the default output of the routine fold( ). There, an opening parenthesis “(“ denotes a base which is paired with a downstream nucleotide, a closing parenthesis “)” a base paired with an upstream nucleotide, and dots denote unpaired nucleotides.

**Figure 1:**
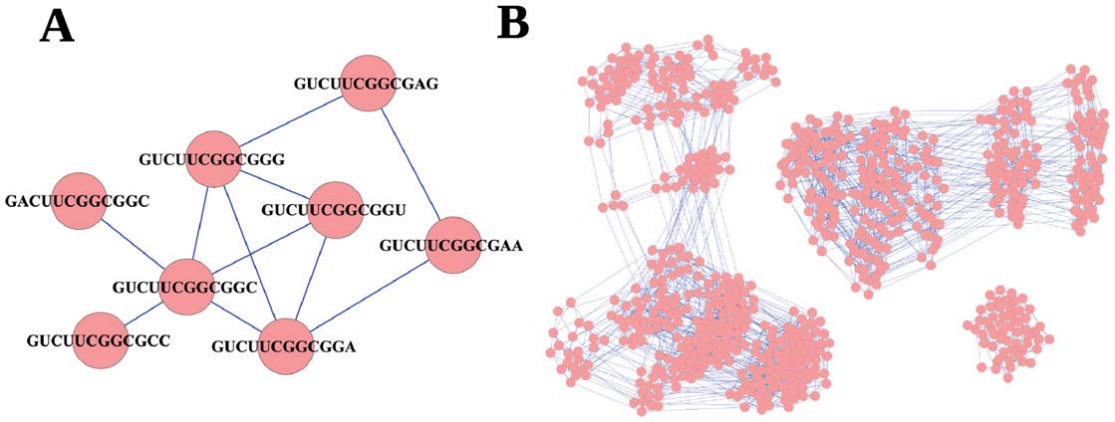
Construction of neutral networks. In (A), we show an example of how neutral networks are constructed: sequences that fold into the same secondary structure are connected if they are at a Hamming distance of one. In (B), we show all sequences of length 12 that fold into the secondary structure (.(....))..., which is ranked in the 46th position. Although all sequences fold into the same secondary structure, the neutral network splits into 3 isolated subnetworks of sizes *N* = 404, 341, and 55.

The 4^12^ = 16777216 molecules fold into 57 different secondary structures plus the open structure, which contains 85% of the sequences. In Table 1, we give all structures, together with the number of sequences folding into each structure (“frequency”). All sequences that fold into the same structure form the neutral network of that structure. By definition, two sequences are linked if they fold into the same secondary structure and differ in a single-point mutation (i.e. they are at a Hamming distance of one, see Fig. 1(A)). Therefore, a neutral network may be connected or disconnected. In the latter case, the neutral network is composed of a number of subnetworks, see Fig. 1(B). For all but two structures, the neutral network is disconnected and formed by 2 to 42 subnetworks, also given in Table 1. In total, 645 different subnetworks have been found for the 57 structures. The open structure (last entry in Table 1) is not considered for the topological analysis.

**Table 1:** Structures and neutral networks obtained from the folding of all sequences of length *l* = 12.

Structures and neutral networks for *n* = 12							
rank	frequency	subnetw.	structure	rank	frequency	subnetw.	structure
1	218567	16	(((....)))..	30	23260	8	...(((...)))
2	183335	10	.(((....))).	31	15350	6	..((......))
3	161765	26	(((.....))).	32	11365	7	...((.....))
4	152393	9	((....))....	33	6940	3	......(....)
5	152221	15	..(((....)))	34	3638	28	((.(....))).
6	121861	8	...((....)).	35	3519	27	(((....).)).
7	117253	21	((((....))))	36	2963	39	((.(....).))
8	113896	8	.((....))...	37	2244	12	(.((....))).
9	110842	22	.(((.....)))	38	2208	1	((........))
10	105538	8	..((....))..	39	1520	16	.(.(....).).
11	93866	7	((.....))...	40	1379	15	(.(....).)..
12	76439	5	..((.....)).	41	1368	2	.((.......))
13	74626	12	(((......)))	42	1308	22	.((.(....)))
14	71904	5	((......))..	43	1189	34	(..(....)..)
15	70375	5	.((.....))..	44	1140	23	.(((....).))
16	61792	7	.((......)).	45	860	3	..(.(....)).
17	61613	27	((((...)))).	46	800	3	(.(....))...
18	46510	10	....((....))	47	713	3	.(.(....))..
19	45288	42	.((((...))))	48	665	15	(.((....)).)
20	41618	18	..(((...))).	49	414	11	..(.(....).)
21	41092	15	(((...)))...	50	314	3	(..(...)..).
22	39740	19	.(((...)))..	51	240	3	(.((...)).).
23	37472	5	((.......)).	52	220	4	((((...)).))
24	31848	3	(....)......	53	211	4	((.((...))))
25	31498	3	.....(....).	54	165	4	..((....).).
26	27522	3	....(....)..	55	153	4	.((....).)..
27	27312	3	.(....).....	56	107	6	(((....)).).
28	25053	3	..(....)....	57	54	1	(.(.....).).
29	24366	3	...(....)...	-	14325304	-	............

Additional properties of the *l* = 12 RNA neutral networks space can be found in [10].

### Definition of topological quantities

Each subnetwork is a connected and undirected graph whose structure is contained in the *adjacency matrix* **A**, with elements *A_ij_* = 1 in case sequences *i* and *j* differ in a single nucleotide, and 0 otherwise.

We compute the *size N*, the total number of links *L* and the *degree distribution p*(*k*), which yields the probability of finding a node of degree *k*, for each subnetwork. The degree corresponds to the number of neighbors *k_i_* of a given sequence *i* within its neutral subnetwork. The local density of links is measured by the *clustering coefficient C*, which is first defined for each node *i* as the probability that two of its neighbors are connected:
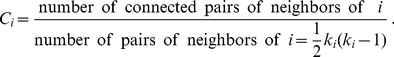
(1)


The *local clustering as a function of degree C*(*k*) is defined as the average of *C_i_* over all nodes with a given degree *k*:
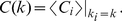
(2)


Finally, the *clustering* of the subnetwork *C* is obtained by averaging over all nodes *C* = <*C_i_*>.

The *shortest path* <*d*> of each subnetwork is calculated as the average of the shortest path length *d_ij_* between any pair of sequences *i, j* belonging to the same subnetwork, 
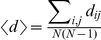
.

The *nearest-neighbor degree k_nn,i_* is another local quantity that measures the average degree of the neighbors of a node *i*. It is usually calculated as a function of the degree *k*,
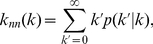
(3)where 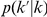 is the fraction of links that are attached to a node of degree *k* whose other ends are attached to a node of degree *k'*. The variation of *k_nn_*(*k*) with *k* is related to the *assortativity* of the subnetwork [32], which indicates the tendency of a node of degree *k* to associate with a node of the same *k*. When *k_nn_*(*k*) is an increasing function, the subnetwork is *assortative* and the most connected nodes (sequences) are prone to be linked to other highly connected sequences. If the *k_nn_*(*k*) function is decreasing, a network is called *dissortative* and indicates that the network hubs are mainly attached to sparsely connected nodes. Assortativity can be quantified by the *degree-degree correlation coefficient r*, which is the Pearson correlation coefficient for the degrees of the nodes at either end of a link:
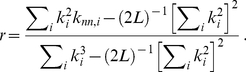
(4)


The *r* parameter and the *k_nn_*(*k*) distribution are closely related: a monotonically increasing (decreasing) *k_nn_*(*k*) corresponds to a positive (negative) value of *r*.

The definition of *betweenness centrality B*(*i*) of a node *i* is given by
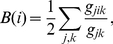
(5)where *g_jk_* is the total number of shortest paths between nodes *j* and *k*, and *g_jik_* is the number of shortest paths between nodes *j* and *k* that pass through node *i*. The *eigenvector centrality v*_1_(*i*) is given by the right eigenvector of the largest eigenvalue *λ*_1_ of the adjacency matrix **A** [33].

Finally, we analyze the community structure of the networks by computing the *modularity Q*, given by [34]:
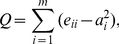
(6)where *m* is the number of communities inside the network, *e_ii_* is the fraction of links in the network connecting nodes of the same community *i*, and *a_i_* is the fraction of links that have one or two ends inside community *i*. Note that the larger the fraction of links inside each community (internal links), the higher the value of *Q*. This way, modularity *Q* is usually taken as the reference parameter in order to find optimal community divisions based on the topological analysis of the networks [35]. In the current work, we have used the extremal optimization algorithm [36] since it has high performance even for networks of large sizes.

### Population dynamics on RNA neutral networks

Though this work is mainly related to the topological description of RNA secondary structure neutral networks, topology becomes especially relevant when one considers the evolution of ensembles of RNA sequences subjected to replication and mutation and suffering the selective pressure of staying on a given neutral network to maintain functionality. Here we introduce the basic rules and quantities related to sequence population dynamics. Select a particular neutral (sub)network and suppose that sequences corresponding to any of the nodes replicate and mutate at each time step. If a mutant coincides with one of the neighboring nodes in the network, its population increases in one unit; if the mutant is not in the network, it is eliminated. This process can be mathematically described as 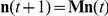, where 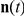 is a vector whose components are the number of sequences at each node of the network at time *t* and **M** is the transition matrix
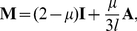
(7)with *µ* being the mutation rate, **I** the identity matrix, *l* the length of the sequence and **A** the adjacency matrix of the network. The eigenvalues *w_i_* of **M** and *λ_i_* of **A** are related by 
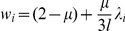
, while both matrices share the same eigenvectors [37].

In the limi *t* → ∞, the population attains a stationary state that is described by the right eigenvector associated to the largest eigenvalue *w*_1_ of **M**, or to the largest eigenvalue *λ*_1_ of **A**. While *w*_1_ yields the growth rate of the population at equilibrium, *λ*_1_ coincides with the *spectral radius* of **A**, which further corresponds to the asymptotic neutrality of the population [38].

## Results

### Neutral network and subnetwork sizes

Table 2 summarizes the main parameters of the space of sequences and neutral networks. In order to compare our results with a randomized RNA neutral network, we have selected at random *N_fold_* sequences from the complete space of length *l* = 12 and connected them if they differ in one position, irrespectively of their corresponding secondary structure. Note that *N_fold_* is the total number of sequences that do fold into a secondary structure, that is, sequences yielding the open structure are discarded. The random network has an average degree 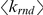 about three times smaller than the average degree 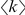 of the real neutral subnetworks. This reveals that neutral networks are not spread over the full space of sequences, but cluster around preferred regions.

**Table 2:** Description of the main parameters of the sequence space.

Parameter	Description	Value
*b*	Number of different bases (alphabet length)	4
*l*	Sequence length	12
*N_total_*	Total number of sequences	4^12^ = 16777216
*N_fold_*	Folded sequences	2451912
*N_struct_*	Number of different secondary structures (networks)	57
*N_net_*	Number of clusters (subnetworks)	645
<*k*>	Average degree of the folded sequences	16.74
<*k_rnd_*>	Average degree of a random network of size *N_fold_*	5.26

<*k_rnd_*> is the expected average degree if the probability of folding into a structure different from the open structure would not depend on the position in the space of sequences.

**Figure 2:**
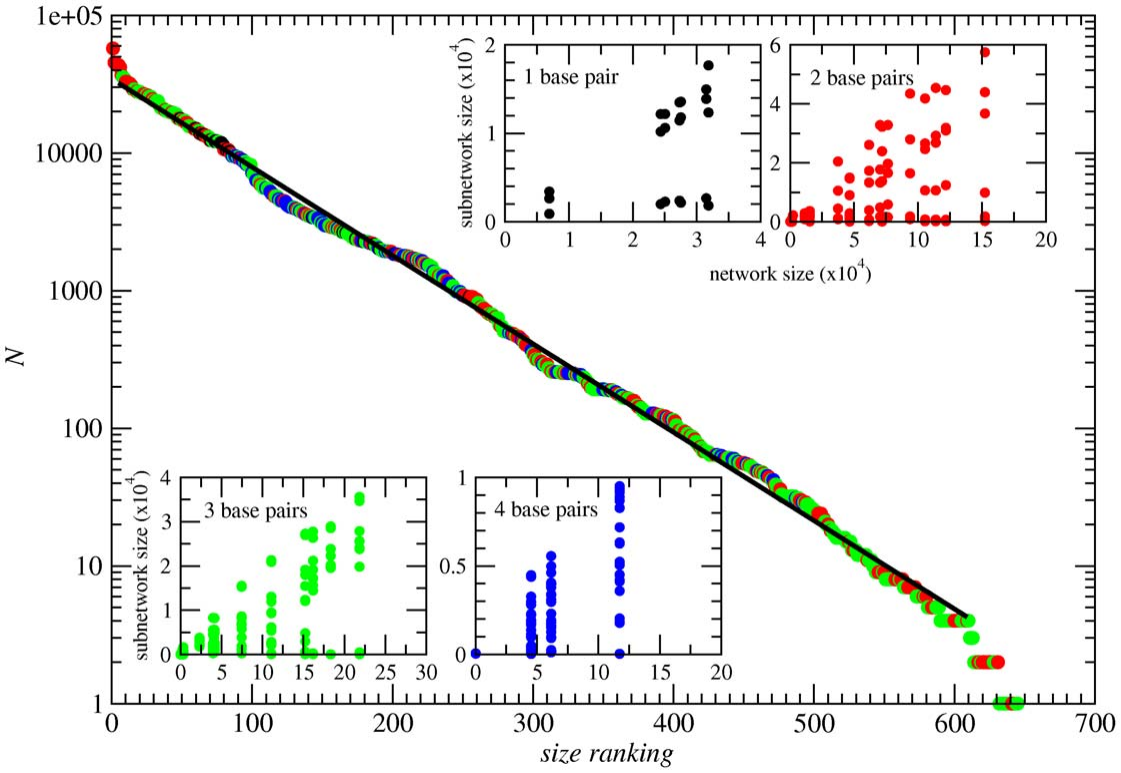
Subnetworks size ranking. In linear-logarithmic scale, ranking distribution of subnetwork sizes. Colors indicate the number of base pairs *L_p_* in the secondary structure: one pair (black), two pairs (red), three pairs (green) and four pairs (blue). The solid line corresponds to an exponential fitting. Insets show for each group of structures (with the same *L_p_*) the size of the subnetworks (in the *y*-axis) that belong to the same neutral network as a function of the corresponding neutral network size (in the *x*-axis). Note changes of scale in both axes.

Figure 2 shows a rank ordering of subnetwork sizes *N*. As a function of rank *r*, they approximately follow 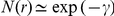, with γ = −0.01515(5). The insets illustrate the relation between such subnetwork sizes and the size of the network they belong to, depending on the number *L_p_* of base pairs in the structure.

Although the five largest networks have a secondary structure formed by only *L_p_* = 2 base pairs, there is no simple correspondence between the number of pairs and the size of the subnetworks. The number of base pairs in the stacks, however, determines the maximum possible number of large subnetworks per structure. Attending to accessibility through point mutations [39], the six possible base pairs can be classified into two groups:
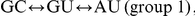


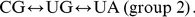
(8)


We will define as *accessible sequences* those whose stacks are identical in composition or differ only in accessible base pairs. Only accessible sequences can belong to the same subnetwork, because base pairs from groups 1 and 2 cannot be connected by single-point mutations. Even when two sequences are accessible, they will only belong to the same subnetwork if there exists a continuous path through sequences belonging to the subnetwork that connects them.

### Degree distributions

The degree of a sequence is a measure of its robustness to mutational changes. The larger its value of *k*, the less likely will be that a random mutation causes a different secondary structure. Degree is thus a first indicator of the functional stability of a given sequence, and by extension of a given secondary structure.

**Figure 3:**
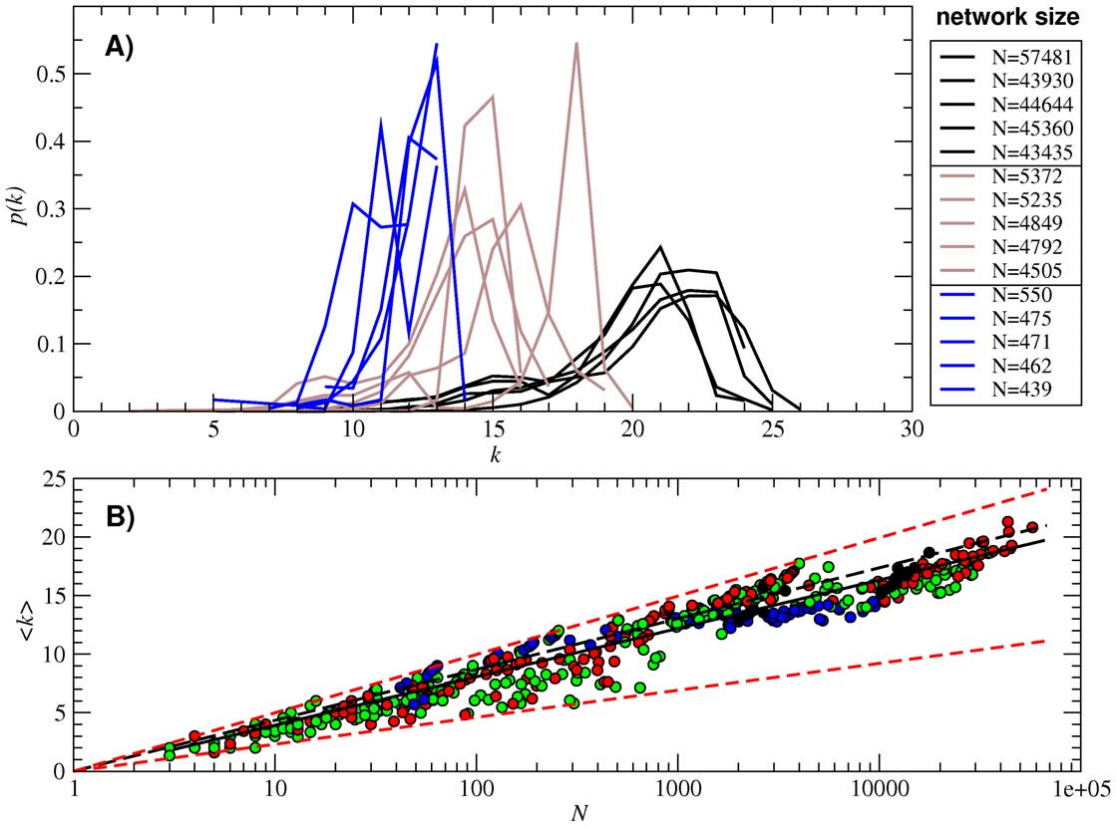
Degree distribution *p*(*k*) and average degree 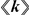 (A) Degree distribution *p*(*k*) of fifteen subnetworks. They are the five largest (black curves), five of intermediate size (brown curves, one order of magnitude smaller) and five small subnetworks (blue curves, two orders of magnitude smaller). (B) Average degree 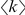 as a function of the subnetwork size *N*. Colors correspond to one (black), two (red), three (green) and four (blue) base pairs in the secondary structure. The solid line corresponds to the numerical fitting 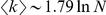 (note the logarithmic-linear scale). The analytical approximation to 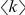 making use of the values of 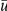, 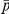 and *α* obtained from all the 12-nt folded sequences (and implying *A_S_* = 0.53) is plotted in long-dashed black line. The upper and lower bounds to coefficient *A_S_* yield 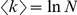 and 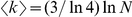 (plotted in short-dashed red lines).

In Fig. 3(A) we plot the degree distribution *p*(*k*) of fifteen subnetworks of different sizes, specifically, the five largest subnetworks (*N*≈5×10^4^) together with five subnetworks that are one (*N*≈5×10^3^) and two (*N*≈5×10^2^) orders of magnitude smaller. These degree distributions cannot be well approximated by any of the usual distributions (such as Poissonian or binomial ones). Nevertheless, they are single-peaked in all cases, with the maximum shifted towards the highest values of the degree. This fact indicates that high-degree nodes are more frequent, despite the cut-off value given by *k_max_* = (*b*−1)*l*, with *b* = 4 the number of different nucleotides and *l* = 12 the sequence length (i.e., *k_max_* = 36). This largest degree is never reached.

Next, we show the dependence of the subnetwork average degree 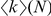 on subnetwork size [Fig. 3(B)]. We observe that the average degree <*k*> grows with size, approximately following 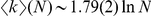. An analogous relationship between neutrality and (estimated) size of a neutral network has been reported in [11].

Attending to some generic properties of the sequence-structure map, it is possible to derive an analytical relationship between the average degree <*k*> and the size of the subnetwork *N*. Generically, a structure is formed by 2*L_p_* nucleotides forming *L_p_* pairs and *L_u_* unpaired nucleotides, with 2*L_p_*+*L_u_* = *l*. Paired and unpaired nucleotides have a different response to mutations, since most neutral mutations, especially for short sequences, occur in unpaired nucleotides [1], [9]. This difference is reduced as the length *l* of the molecule grows. In the limit of large *l*, the probability of the paired nucleotides supporting neutral mutations in an RNA molecule and the corresponding value for unpaired nucleotides become independent of the length *l* [7], [8].

We denote by *p*−1≥0 the average number of neutral mutations per base pair that a given sequence can accept and by *u*−1≥0 the corresponding average number of neutral mutations per unpaired nucleotide. The values of *u* and *p* are bound due to the size of the alphabet and the possible chemical interactions between nucleotides, such that *u*≤4 and *p*≤3. Given *u* and *p* for a sequence, its degree can be obtained as *k* = *k_p_*+*k_u_*, with *k_p_* = (*p*−1)*L_p_* and *k_u_* = (*u*−1)*L_u_*. These quantities can be further averaged over all sequences belonging to the same neutral (sub)network, such that its size can be estimated as
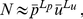
(9)where 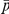 and 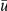 count the actual average number of pairs and nucleotides at paired and unpaired positions, respectively, that maintain the secondary structure (see also [8]). Clearly, *N* is a structure-dependent quantity. For later convenience, let us now define
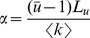
(10)as the average fraction of total mutations that occur in unpaired nucleotides for a given structure. Simple algebra leads to
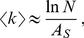
(11)with
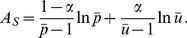
(12)


*A_S_* depends implicitly on 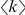 through *α*. Substituting this expression in Eq. (11) and developing in powers of 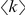, we obtain
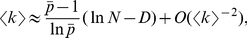
(13)where 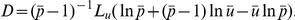 Therefore, the main order in 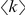 yields the expected functional form 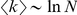. According to their definition, parameters 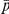 and 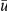 depend on the structural state of a nucleotide (whether paired or unpaired), and as such are mostly independent of the particular structure considered. However, *α* contains explicit information on the number of unpaired (or paired) nucleotides in a structure, and hence is a structure-dependent quantity (in fact, it is clear from Eqs. (10) and (13) that *α* decreases with *N* and with the number of base pairs *b*). This implies that there is an intrinsic dispersion in the values of the average degree due to the structure-dependent coefficient in Eq. (11). This dispersion is clearly visible in Fig. 3(B), where each point corresponds to one of the 645 subnetworks and where no statistical errors are present. The extreme values of *A_S_* can be however obtained (and the corresponding approximations for 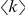 are plotted in short-dashed red lines). The maximum value of *A_S_* is one, and it is obtained when any mutation destroys the secondary structure considered (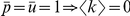). This is however a marginal case where *N* = 1 by definition. Values of *A_S_* close to one are only possible for very small networks. The function 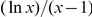 is monotonically decreasing. Hence, the minimum value of 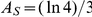 is attained when all mutations occur in unpaired nucleotides (independently of their precise number) and any mutation is accepted, such that *α* = 1 and 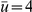. Furthermore, a more precise value of *A_S_* for our case can be calculated by making use of the numerical estimations for 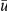, 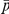 and *α* obtained as the average of all 12-nt folded sequences. This calculation yields 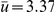, 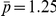, *α* = 0.95 and *A_S_* = 0.53 (long-dashed black line in Fig. 3(B)). Note that, for this calculation, we have assumed an average, constant *α* for all structures. Other values previously reported in the literature for *α* also show that the fraction of total mutations that lies on the unpaired nucleotides is close to 1, such as for example *α* = 0.84 for the 76-nt tRNA molecule [1], [9].

### Clustering

The clustering coefficient *C* quantifies the amount of links existing between the neighbors of a given sequence. It is a measure of cliquishness [32] that reveals deviations from random relationships between nodes. Usually, low values of *C* correspond to randomly connected networks, while values above the random expectation indicate the existence of local correlations and, in the case of RNA neutral networks, the presence of regions in sequence space which are more robust than average with respect to mutations.

**Figure 4:**
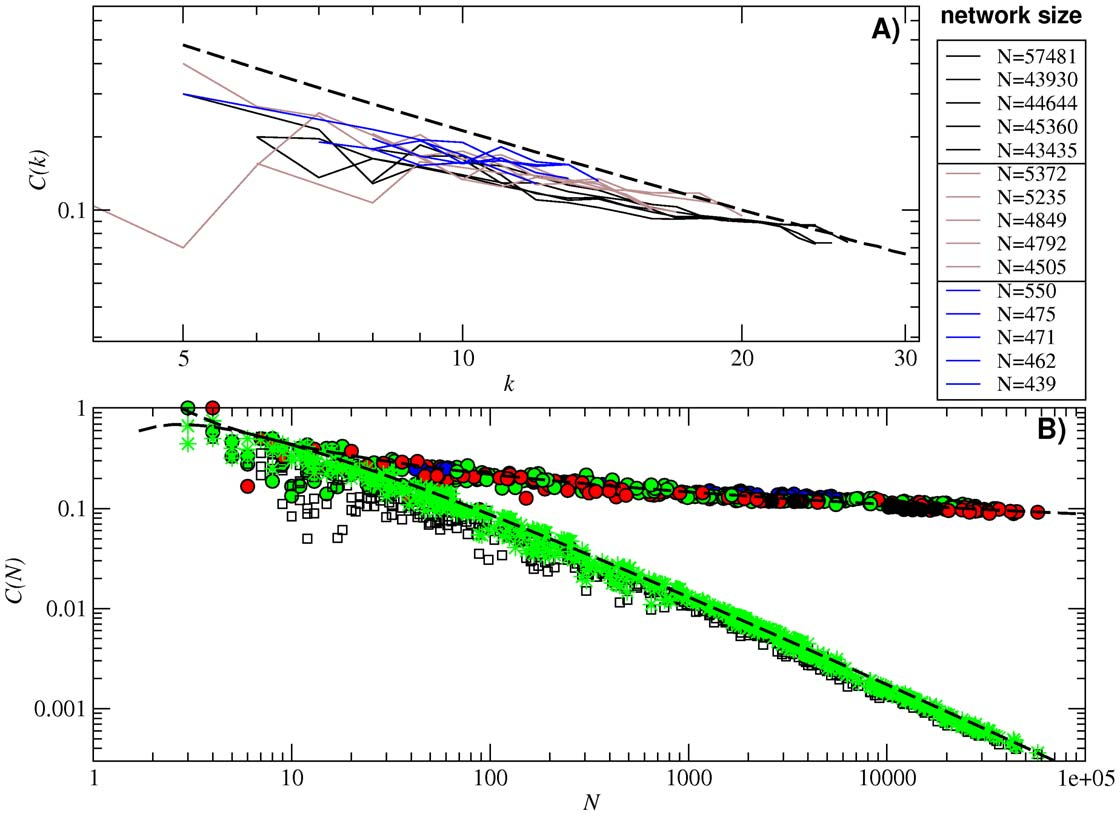
Clustering. (A) Clustering distribution *C*(*k*) for the fifteen networks analyzed in Fig. 3. (B) Average clustering *C*(*N*) as a function of the subnetwork size *N* for all folded neutral networks (colored circles), equivalent random networks (black squares) and theoretical predictions with a classical random model (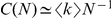, green stars). Circle colors correspond to the number of base pairs of each subnetwork (see caption of Fig. 3). In both plots (A) and (B), the analytical approximations using the values of 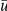, 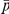 and *α* obtained from all the 12-nt folded sequences are plotted in long-dashed black lines.

Figure 4(A) shows the clustering coefficient *C*(*k*) as a function of the degree *k* for the previously analyzed subnetworks. It suggests that data are compatible with a power-law decay of the form *C*(*k*)∼*k* ^−1^, regardless of the subnetwork size. This scaling has been previously reported in other kind of biological networks, such as metabolic [40] and protein networks [20], and has been usually attributed to their hierarchical modularity. In those networks, sub-modules integrate, at different scales, into larger modules [40], leading to the observed power-law decay of the clustering. However, this functional behaviour of the clustering with the degree can only be obtained if the degree distribution has a scale-free structure *p*(*k*)∼*k* ^−*γ*^. This is not the case of neutral networks, where the power-law decay of the clustering distribution is related to the structural properties induced by folding and to the alphabet size.

The numerical dependence of the average clustering coefficient *C*(*N*) on the subnetwork size *N* is shown in Fig. 4(B). In order to evaluate the degree to which our networks depart from their randomized counterparts, we compare the *C*(*N*) distribution with the one obtained in equivalent random networks. The latter networks have been obtained by randomly reshuffling the links within each subnetwork, disregarding biological constraints, but keeping the degree distribution *p*(*k*) fixed (black squares of Fig. 4(B)). Note that this operation destroys the geometrical structure underneath the networks, despite the fact that each sequence (node) maintains its number of neighbors. The result is that the clustering distribution of neutral networks is not similar to that of usual random networks, for which 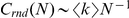 holds [41] (green stars of Fig. 4(B)).

Applying some simple assumptions, and making use of Eq. (11), we can obtain analytical expressions for *C*(*k*) and *C*(*N*). Nucleotides forming pairs cannot contribute to clustering, since at most one mutation can be accepted without breaking the pair: a nucleotide in a stack can have at most degree one. All triangles are thus contributed by unpaired nucleotides accepting two or three mutations. For a given sequence *i*, Eq. (1) implies 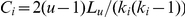. Averaging over all sequences with *k* neighbors and using the definition of *α*, we obtain
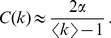
(14)


Direct substitution of (11) into (14) yields the dependence of the average clustering coefficient *C*(*N*) on the subnetwork size,
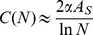
(15)for large values of *N*. For random networks, it becomes
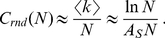
(16)


The analytical approximations above are compared to our numerical results in Fig. 4.

### Assortativity

Another indicator of the local organization of a complex network is the average-neighbor degree *k_nn_*(*k*), which relates the degree *k* of a node with the average degree of its neighbors. In random networks, *k_nn_*(*k*) and *k* are not correlated. In most biological networks the average degree of the nearest neighbors is negatively correlated with *k* (examples are genetic, protein and metabolic networks [16], [23], [42]), with the only known exception of fMRI functional brain networks [43].

**Figure 5:**
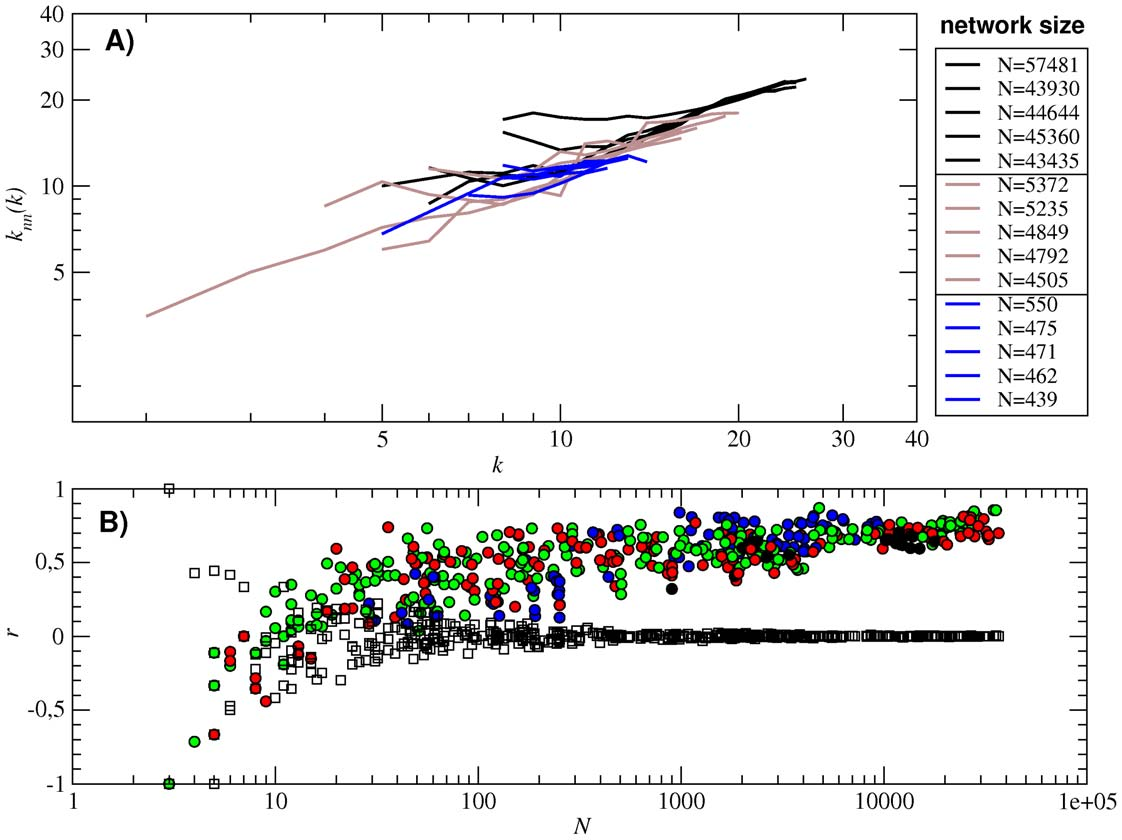
Assortativity. (A) Average nearest neighbors degree *k_nn_*(*k*) as a function of *k* for fifteen networks of different sizes. (B) Assortativity parameter *r* as a function of the network size. As in previous figures, colors correspond to the number of base pairs of the subnetwork: one (black), two (red), three (green) and four (blue). The *r* for equivalent random networks are plotted in black squares.

Figure 5(A) shows the function *k_nn_*(*k*) for the fifteen networks previously analyzed. In all cases, we obtain a dependence compatible with an algebraic growth, *k_nn_*(*k*)∼*k^β^* with *β*≈0.75, which indicates a positive correlation between the degree of a node and the average degree of its neighbors. In other words, nodes with high degree are prone to be connected between them. Networks with this kind of local organization, which are called *assortative* [23], are more robust against disconnection processes due to the fact that network hubs are linked together forming high-degree cores.

In Fig. 5(B) we analyze the dependence of assortativity on subnetwork size by measuring the assortativity parameter *r*. With the exception of small subnetworks (with less than ten nodes, approximately) all subnetworks have an *r* parameter higher than zero, i.e., they are assortative [32]. In addition, *r* on average increases with the network size *N*, which indicates that, the larger the network, the higher the cohesion between high degree sequences. Equivalent random networks generated as explained in the previous section yield *r*→0 for *N* sufficiently large, as expected.

**Figure 6:**
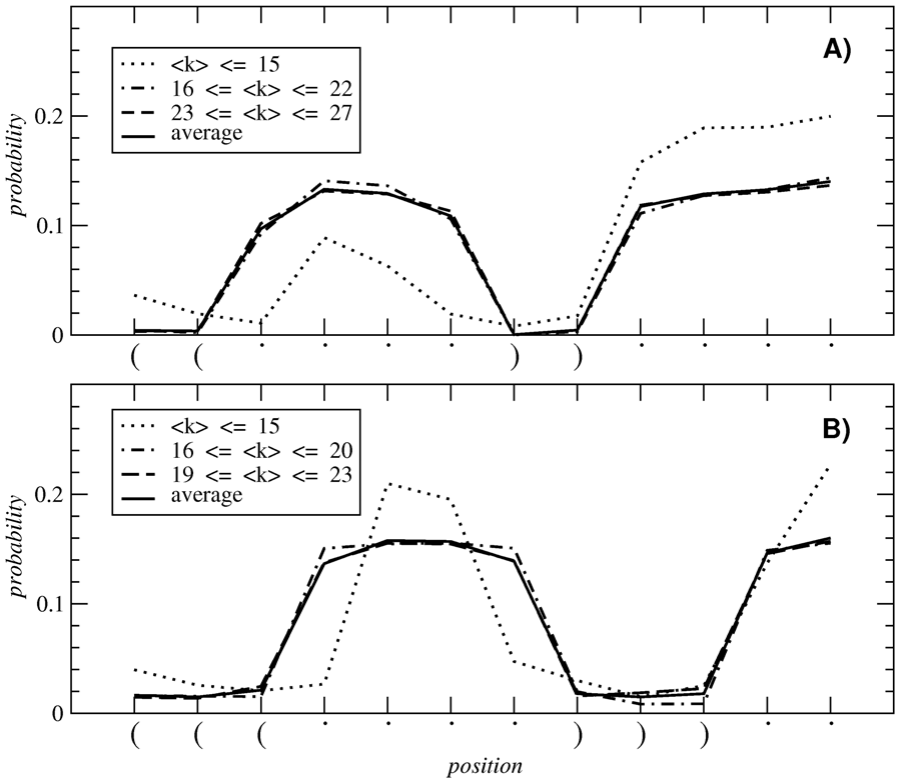
Probability of mutation. Probability of mutation at each position of the sequence for two different secondary structures (see *x*-axis labels of both plots). (A) corresponds to the largest subnetwork *N* = 57481, whose secondary structure is fourth by abundance. (B) corresponds to the largest subnetwork *N* = 35594 of the most abundant secondary structure. We plot the sequences grouped by degree (dotted, dashed and dashed-dotted lines) together with their averages (solid lines).

The assortativity of RNA neutral networks can be explained by analyzing how the probability of a neutral mutation depends on the position in the sequence. Figure 6 shows the probability that a sequence mutates at each of its *l* = 12 positions without disrupting the secondary structure. Two examples are shown: the case of the largest subnetwork in Fig. 6(A), and the case of the largest subnetwork of the most abundant secondary structure, in Fig. 6(b). As discussed, most mutations occur in unpaired nucleotides [9], since base pairs are the main contributors to the stability of the secondary structure. Thus, sequences that have strong base pairs will support a higher number *u* of neutral mutations, forming high-degree nodes. In addition, neighbor sequences of the highest degree nodes will maintain the base pairs (and the energy associated to them) and therefore they will also be high degree nodes, leading to an assortative configuration. Since high-degree nodes on average have lower folding energy, this can be associated to the correlation between the neutrality and the thermodynamic stability of sequences already described in RNA [44].

### Average shortest path

A first quantification of the navigability of neutral networks is yielded by the average shortest path between any pair of nodes. Since RNA neutral networks are embedded in regular lattices of very high dimensionality (actually, of a dimension equal to the length of the sequences *l*), the distance between an arbitrarily chosen pair of sequences in a subnetwork could be extremely large if only point mutations are allowed. In fact, an exact calculation of the longest path for a hypercube of dimension *l* = 12 (i.e. the still open snake-in-the-box problem for an alphabet of 2 letters [45]), is 1260 [46]. In a 4-letter alphabet this quantity will be significantly higher, though analytical estimates are not currently available. In order to check whether neutral networks show such long distances linking some of their nodes, or on the contrary resemble in some way small-world networks [23], [33], [47], we have calculated the average shortest path 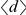 in each subnetwork.

**Figure 7:**
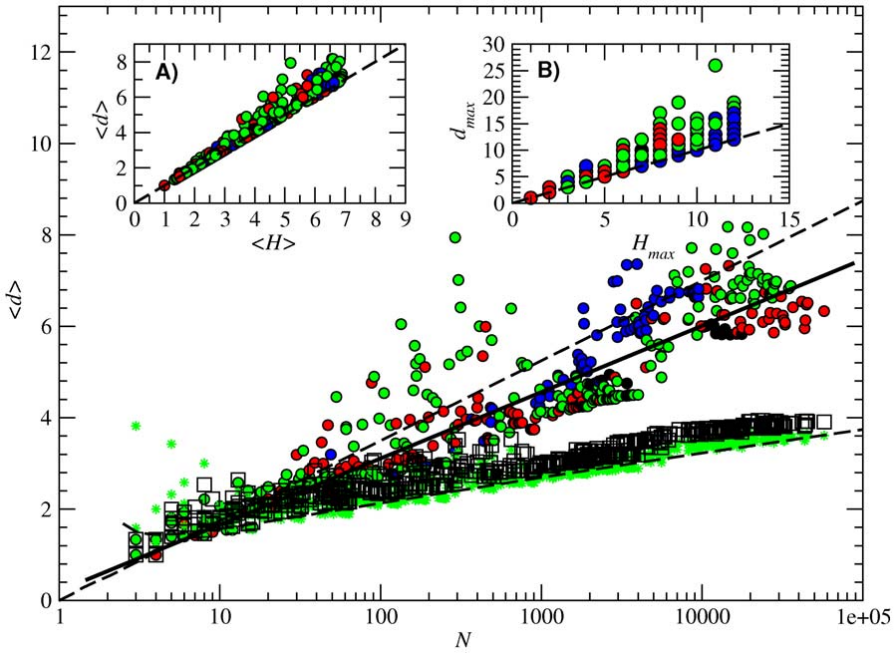
Average shortest path 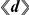 Dependence of the average shortest path on the subnetwork size *N* for all folded neutral networks (colored circles), equivalent random networks (black squares) and theoretical predictions with a classical random model (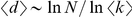, green stars). Circle colors correspond to the number of base pairs of each subnetwork (see caption of Fig. 3). The numerical fitting is plotted as a solid black line, while the analytical approximations correspond to the long-dashed black lines (for values of *α* and *A_S_* numerically obtained from the folding of all 12-nt sequences). Inset (A): relation between the average shortest path 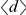 and the average Hamming distance 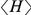 of the subnetworks. Inset (B): relation between the longest distance between any pair of nodes of the network *d_max_* and the maximum number of different bases between sequences *H_max_* (maximum Hamming distance). In the insets, the dashed lines are 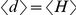 and 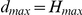, which correspond to the lower bounds of 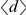 and 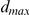, respectively.

Small-world networks are characterized by a high clustering coefficient *C* (when compared to an equivalent random graph) and low average shortest path between nodes (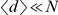). As we have seen, RNA neutral networks fulfill the clustering requirement; in Fig. 7 we now show that, despite the fact that the average shortest path 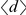 varies with the network size, its functional dependence is far from that expected in random networks: the average shortest path scales in our case with the logarithm of the network size [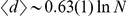, solid black line in Fig. 7], while the shortest path of the equivalent random networks is close to the analytical prediction 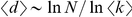 [41] (green stars). In inset Fig. 7(A) we plot the relation between the shortest path length 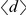 and its lower bound, the average Hamming distance 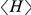 of each subnetwork. Both values are very close, independently of the size of the subnetwork. Something similar happens to the diameter of the network *d_max_* (number of steps between the most distant nodes), which remains remarkably close to its lower bound *H_max_* (inset Fig. 7(B)).

The previous results can be explained in the light of some properties of RNA neutral networks. According to our previous numerical results and some heuristic reasoning already presented, most sequences within a given subnetwork differ mainly in the unpaired nucleotides, while all 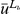 sequences sharing the same base pairs will belong to the same subnetwork. Following these hypotheses, and taking into account that measured 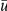 for most structures yield values close to their upper bound 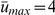, it is straightforward to see that the distances between the nodes that share the same base pairs will be similar to their Hamming distance, and therefore we can approximate the average distance in a subnetwork to 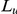. Properly, this quantity is a lower bound for the maximum distance 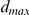 in the subnetwork, since mutations in the stacks are also possible. Assuming that 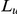 is an acceptable approximation for the average distance 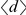, we obtain
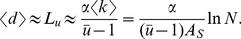
(17)


The average distance for the randomized networks reads
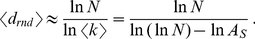
(18)


Once more the functional dependence is correctly recovered via a simple analytical treatment (see Fig. 7).

### Sequence Centrality

Centrality, as its name suggests, is a measure that differentiates nodes according to how influential, or central, they are in a network. The degree *k* of a node is a first indication of its centrality, since it is intuitively reasonable to assume that sequences with a high degree will be traversed by a proportionally larger number of shortest paths. However, the degree is a local measure, since, among others, it does not take into account the importance of the neighbors of a given node. To overcome this restriction, centrality can also be estimated through different non-local quantities, such as closeness, betweenness, and eigenvector centrality [33]. Among them, we have chosen the *eigenvector* and *betweenness* centrality, since they are related to population dynamical processes that may occur on the neutral networks.

**Figure 8:**
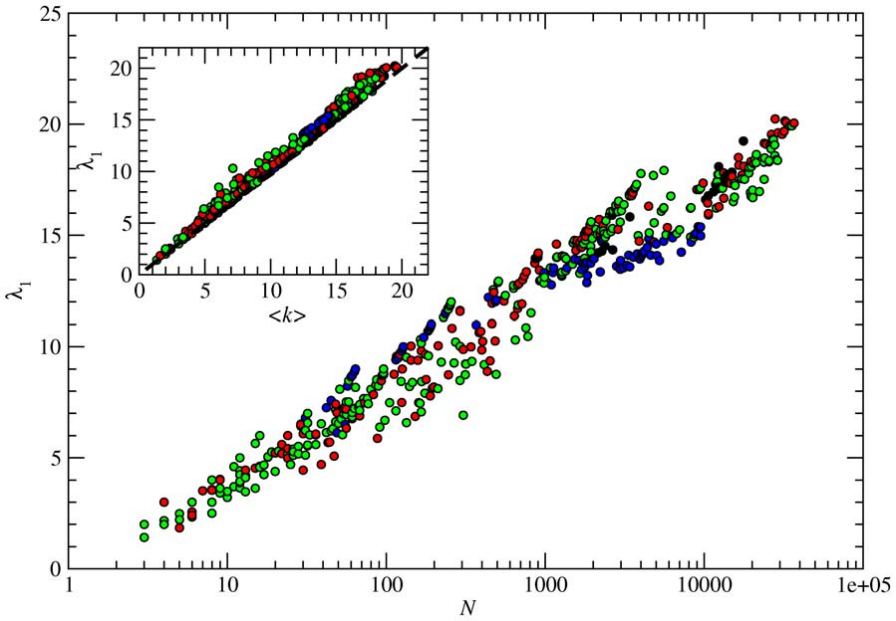
Eigenvector centrality. Largest eigenvalue *λ*_1_ of the adjacency matrix **A** as a function of the network size *N*. The inset shows the linear relationship between *λ*_1_ and the network average degree 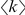. Solid line in the inset is 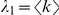.

Eigenvector centrality is a particularly interesting measure in our kind of networks, since it coincides with the fraction of population (number of genotypes of each sequence) at stationarity under replication and mutation on the network [37], [38]. In addition, the largest eigenvalue *λ*_1_ of the adjacency matrix **A** gives the average degree of the population (see the last subsection of the Methods for more details). The relation between *λ*_1_, the subnetwork size *N* and the average degree 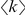 of the subnetwork is shown in Fig. 8. *λ*_1_ depends logarithmically on *N*, due to the fact that the network average degree 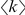 and *λ*_1_ are linearly correlated (inset), always fulfilling that 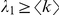 [38]. In other words, the population concentrates in regions of the network with a connectivity above average, thus increasing its robustness to mutations.

**Figure 9:**
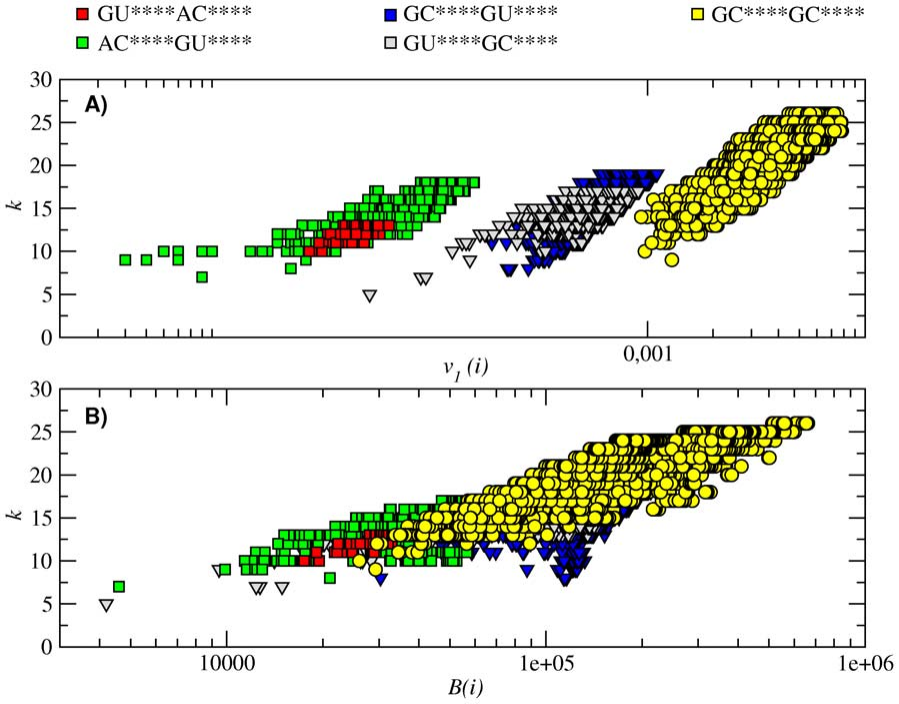
Sequence centrality. Evaluation of the sequence centrality for the largest subnetwork *N* = 57481, whose secondary structure is ((....))..... In (A), degree *k_i_* versus eigenvector centrality *v*_1_(*i*). In (B), degree *k_i_* versus betweenness centrality *B*(*i*). Colors and shapes denote the type of base pairs the sequences have (see Figure's legend). Note the community division created by the eigenvector centrality, which is related to the type of nucleotides participating in the base pair: GC+UA and AU+CG for low eigenvector centrality, GU+CG and GC+UG and for intermediate *v*_1_(*i*) and GC+CG for high *v*_1_(*i*).

The betweenness of a node *B*(*i*)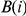 quantifies the probability that node *i* represents an intermediate step in the evolution of the population from one sequence to another. Figure 9 shows the relation between the degree of the sequences *k_i_* (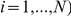 and (A) the corresponding component of the eigenvector *v_i_*, and (B) the betweenness centrality *B*(*i*) for the largest subnetwork (*N* = 57481). In Fig. 9(B), we observe a positive correlation with the degree, which confirms the intuitive idea that sequences with higher degree are those with higher betweenness: the larger the number of neighbors of a given sequence, the higher the probability of being in the mutational path between two other sequences. Deviations from this correlation would indicate an “anomalous” distribution of hubs (e.g., hubs placed at the corner of a network). While we have found that for this network the eigenvector centrality is approximately proportional to the betweenness, in this case the former quantity is more informative than the latter. Already at first sight [Fig. 9(A)], we observe a division of the subnetwork into three well-defined communities, each of them corresponding to a certain base pair present (AU, GU, or GC), in addition to a GC pair which is always found. From left to right, the communities increase their size (number of nodes in the community) and also the population per node. Inside each community, the eigenvector centrality shows a correlation with the sequence degree, revealing that high degree nodes are those with higher centrality. Nevertheless, since the division in communities is a consequence of almost one order of magnitude difference in the eigenvector centrality, it is not only the degree of the sequence, but also the community where the sequence belongs to, what determines the population of a node in the subnetwork. It is worth comparing the division into communities given by the first eigenvector with that obtained with classical community division algorithms [35], which split a network by optimizing the modularity *Q* and only taking into account the topological information (disregarding, e.g., that certain base pairs are conserved within the same subnetwork). We obtain a value of *Q* = 0.177 for the eigenvector partition and *Q* = 0.626 for an optimal partition given by the extremal optimization algorithm [36]. Nevertheless, the latter topological division, which splits the network into *m* = 19 communities, contains sequences with different base pair composition within the same community, which hinders the biological interpretation. Further work analyzing the interplay between the partitions obtained by modularity optimization and those given by the base pair composition should be addressed in the future.

### Percolation transitions

A random counterpart of RNA neutral networks is represented by random geometric graphs (RGG) [48], [49], whose nodes sit in a space embedded with a measure of distance. Two nodes are connected if their distance is below a given threshold. There exists a value of this distance (related to the average degree of the nodes) where initially isolated graphs coalesce to form a unique giant component in a percolation transition. Below this transition, the degree distribution is peaked at a well-defined average value with a finite variance, similar to the distribution observed for Erdös-Renyi (ER) random graphs (where, however, no measure of distance is defined). RNA neutral networks present a comparable distribution of degrees (Fig. 3). The geometrical nature of RGG, where nodes are connected depending on their distance, gives rise to structures with much larger clustering coefficients and average path lengths (the latter due to the absence of shortcuts between distant nodes) than those of typical Erdös-Renyi random graphs [50]. The exponentially decaying rank-ordering of network sizes shown in Fig. 2 resembles that of random graphs that are well above or below the percolation threshold [41] or that of random geometric graphs (RGG) below the critical connectivity [48]. These percolation transitions are ubiquitous in systems where an ensemble of nodes is linked through a variable number of connections. Actually, the transition has been studied in RNA neutral networks and has been shown to depend on the size of the alphabet of nucleotides and on the length of the sequences [4], [7], [8]. The case we are studying in this contribution is on average below the percolation threshold, which in turn implies an exponentially decaying distribution of (sub)network sizes. However, the transition to percolation also depends on the average degree 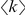 of a graph, and we have observed that our largest networks (which have the largest average degree by virtue of the positive correlation between the two variables) experience a sort of coalescing transition. This is observed in the insets of Fig. 2, where there is a “critical” connectivity above which the subnetworks become connected (except for symmetry properties that prevent accessibility). This critical connectivity is related to the values of 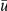 and 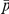 of those particular structures, which may put them above the percolation threshold [4].

**Figure 10:**
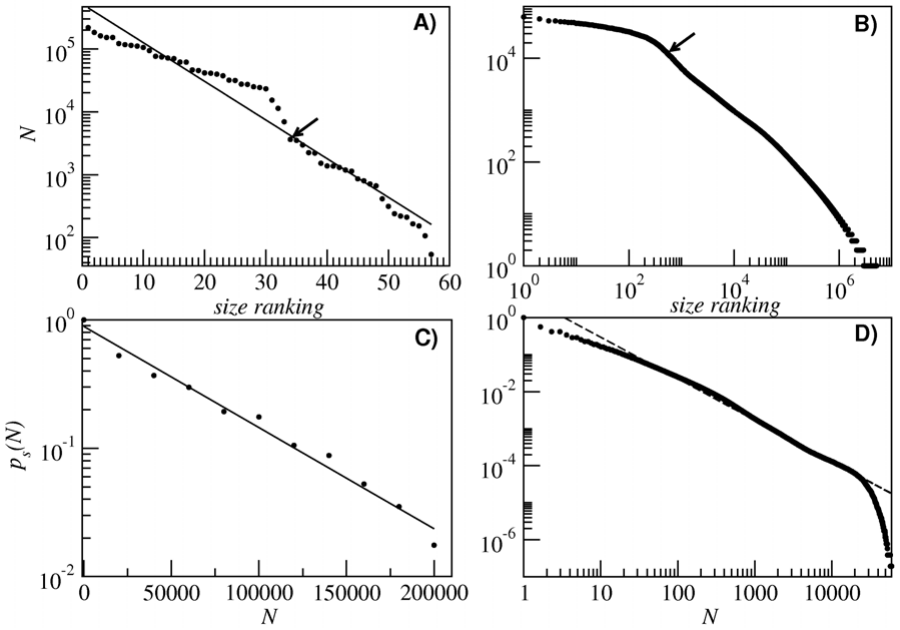
Comparison between *l* = 12 and *l* = 35 neutral networks. Rank ordering of network sizes for *l* = 12 (A) and *l* = 35 (B). Black arrows signal the first non-stem-loop structure. Network size abundance for *l* = 12 (C) and *l* = 35 (D). The solid lines correspond to exponential fits, while the dashed line corresponds to a logarithmic decay. Data for *l* = 35 after [13].

It might be of interest to compare the present results with an extended (though not exhaustive) study carried out for *l* = 35. Figure 10 shows a comparison between the *l* = 12 case and a sample of 10^8^ sequences of *l* = 35 studied in [13]. We have plotted the size ranking for the 57 secondary structures with *l* = 12, Fig. 10(A), and for the 5163323 structures detected with *l* = 35, Fig. 10(B). In the first case, and despite the fact that we have added up all subnetworks corresponding to the same structure into a unique (fragmented) network, we still see an exponential decay. In the *l* = 35 case this curve has a much longer and fat tail (see [13] for a detailed explanation of its nature and the differences with a power-law curve). It is remarkable that in both cases the most abundant structures are of the stem-loop type, that is, they are formed by a loop, a unique stack, and perhaps one or two dangling ends (the black arrows in Fig. 10 point out the first structure that is not of the stem-loop type). Figures 10(C) and (D) show the cumulative abundance of the networks depending on their size. In the case of *l* = 12, the decay is again exponential while for *l* = 35 the decay is logarithmic (with exponent *γ*∼−2 in the non-cumulative curve) and shows a sharp decay for high sizes, just as it happens for random geometric graphs around the critical connectivity [48].

## Discussion

RNA neutral networks are strongly constrained by energetic and structural restrictions inherent to folding. As a consequence, the topological structure of these networks significantly deviates from those of regular or random graphs, and also from the structure observed in other biological networks. With the aim of characterizing the topological signatures of RNA neutral networks, we have analyzed the connected (sub)networks obtained from the folding of the full space of sequences of *l* = 12. We have obtained 57 different secondary structures (i.e., 57 neutral networks), but as most networks are fragmented, our analysis has been directed to the 645 different neutral subnetworks. Although the numerical folding of the RNA sequences is very complex and takes into account many experimentally measured parameters, simple assumptions about how the neighborhood of single structures is conditioned by its structural elements have allowed us to obtain precise analytical approximations for the functional relations between the main topological properties of the networks. Our analytical results do not depend on the length of the sequence, so they should hold generically for all RNA secondary structure neutral networks.

An important feature that distinguishes RNA neutral networks from their random counterparts (Erdös-Renyi random networks and random geometric graphs) is the dependence of the average degree 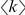 on the size of the subnetworks: 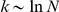. Neutral networks also present the two characteristics that define small-world networks: they have a high clustering coefficient (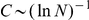), just as typical RGG, but a very low average shortest path between nodes (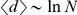), contrary to the expectation in RGG. Note that neither RGG nor neutral networks have *bona fide* short-cuts as it occurs in ER random networks, where no distance can be defined. Nevertheless, the largest distance between two sequences in a neutral network is larger than but close to its Hamming distance, which, in turn, is bounded by the alphabet size *b* and the sequence length *l* as 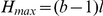. This upper bound for the Hamming distance, which does not exist in RGG, permits a low average shortest path even for large network sizes.

It might be clarifying to comment on the structural differences between RNA neutral networks and other well-known networks. In Table 3 we summarize the differences with two classical network models and in Table 4 we do the same with other biological networks. Neither the classical random model, given by Erdös and Renyi, nor the scale-free model, introduced by Barabási and Albert, reproduce the topological structure of neutral networks. The main discrepancy arises in the logarithmic relation between the average degree 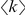 and the size of the subnetwork. This dependence affects the clustering coefficient, which shows a slow decay with the network size, 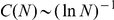. Other folding constraints are reflected in an average shortest path that verifies 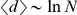 and is above that obtained in both theoretical models, as a result of geometrical constraints imposed by the underlying lattice structure. Finally, neither the classical random model nor the scale-free model can describe the assortative configuration of the nodes.

**Table 3:** Comparison of neutral networks of *l* = 12 with classical random and scale-free networks.

	Neutral Networks (*l* = 12)	Random (Erdös-Renyi)	Scale-Free (Barabási-Albert)
*p*(*k*)	single-peaked	Poisson distribution	power law (∼*k* ^−3^)
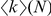	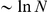	constant	constant
*C*(*k*)	∼*k* ^−1^	constant ( 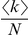 )	constant (∼*N* ^−0.75^)
*C*(*N*)	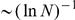	∼*N* ^−1^	∼*N* ^−0.75^
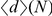	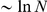	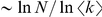	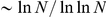
*k_nn_*(*k*)	∼*k* ^0.75^	constant ( 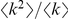 )	non trivial [51]
Assortativity	assortative (*r*>0)	not assortative (*r*→0)	not assortative (*r*→0)

**Table 4:** Comparison of neutral networks of *l* = 12 with other types of biological networks.

	*p*(*k*)	*C*(*k*)	*k_nn_*(*k*)	*r*
Neutral netw. (*l* = 12)	single-peaked	∼*k* ^−1^	∼*k^δ^*	*r*>0
Metabolic networks	PL [20], [22], [26], [40]	∼*k* ^−1^ [40]	NC	*r*<0 [23]
Protein networks	PL [19], [21], [52], [53]	∼*k* ^−2^ [20]	NC [22], PC [54]	*r*<0 [21], *r*>0 [54]
Brain functional netw.	PL [43]	PC [55]	PC [55]	*r*>0 [43]
Ecosystems (foodwebs)	PL [56]–[58], TPL [57], [58], E [58]	–	–	*r*<0 [59], [60]

Some examples of network parameters in different biological networks: degree distribution *p*(*k*), clustering distribution *C*(*k*), degree-degree distribution *k_nn_*(*k*) and assortativity parameter *r*. Abbreviations correspond to: power law ∼*k^γ^* (PL), truncated power law 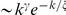 (TPL), exponential 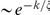 (E), positive correlation (PC) and negative correlation (NC).

The comparison between neutral networks and other biological networks (Table 4) is more difficult since studies where a group of networks of different sizes have been analyzed are rare. Therefore, we are bound to compare network properties that do not depend on network size. At odds with what is found in metabolic, protein or brain functional networks, the degree distribution is not a power law, but has a well defined average, with a maximum value *k_max_*. Concerning the clustering coefficient, we obtain a power-law decay with 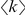 and exponent *γ* = −1 as in metabolic and protein networks. Nevertheless, the origin of this scaling is again a consequence of folding constraints and does not rely on hierarchical modularity, as it occurs in metabolic networks [40]. As it happened with the theoretical models, the assortative nature of neutral networks does not fit with the general assumption that biological networks are dissortative. Nevertheless, we have explained how the dependence of the probability of mutation on the position of the sequence makes high degree nodes to be connected between them. This property, which does not apply for protein, metabolic or genetic networks, is the origin of assortativity in neutral networks, and together with the other topological and statistical properties discussed make of RNA neutral networks a new kind of natural networks.

Community structures in RNA neutral subnetworks can be extracted by the inspection of the first eigenvector of the adjacency matrix, which, in turn, is associated with the final distribution of the population after an evolutionary process [37]. This way, networks present moderate modularity *Q*, being each community characterized by the base pair combinations present in the stacks. Taking into account that the most stable pairs are GC (or CG), followed by AU (or UA) and finally GU (or UG), we have seen that sequences with the most stable stacks will be the most abundant and the most populated in each subnetwork, as their robustness will permit more mutations in the unpaired bases. Further studies on the community structure of these networks and its relevance in dynamical processes are left for the future.

The topological properties of RNA neutral networks have important consequences for the evolution of sequence populations across the space of genomes. Our results give an additional reason to explain the observation that common RNA structures seem to be the ones present in natural, functional RNA molecules [5], [14]. Certainly, as it has been argued, the fact that they are more abundant is a first straight reason for their preeminence [10], [11] though, at equal abundance, networks can still have very different attainabilities [12]. Here we have shown an additional fact, that is, that more abundant structures are those with the highest average connectivity. As a consequence, abundant structures are embedded with a larger-than-average neutrality, such that large neutral networks also offer a robustness to mutations above that of neighboring (but less abundant) structures. For all other parameters being identical, a high average connectivity diminishes the fragmentation of the neutral network and thus facilitates the navigation of the space of genomes and the finding of RNA structures with new functions.
