# Correction: Topological Structure of the Space of Phenotypes: The Case of RNA Neutral Networks

**DOI:** 10.1371/annotation/fb422230-9702-4eed-ad96-1c4cd2ab5e5d

**Published:** 2011-12-13

**Authors:** Jacobo Aguirre, Javier M. Buldú, Michael Stich, Susanna C. Manrubia

There was a typographical error in the first sentence in the second paragraph under the "Population dynamics on RNA neutral networks" heading. "limi" should be "limit".

