# Correction: Topological Structure of the Space of Phenotypes: The Case of RNA Neutral Networks

**DOI:** 10.1371/annotation/b3e79f42-7316-4ff8-9762-514120463813

**Published:** 2011-12-13

**Authors:** Jacobo Aguirre, Javier M. Buldú, Michael Stich, Susanna C. Manrubia

There was an error in the text "B(i)B(i)" in the third paragraph under the "Sequence Centrality" heading. The correct text is "B(i)".

