# Correction: Topological Structure of the Space of Phenotypes: The Case of RNA Neutral Networks

**DOI:** 10.1371/annotation/0a0bed45-e421-4f6a-8d7f-f48194a14516

**Published:** 2011-12-13

**Authors:** Jacobo Aguirre, Javier M. Buldú, Michael Stich, Susanna C. Manrubia

There was a spelling error in affiliation 1. The correct affiliation 1 is "Centro de Astrobiología, CSIC-INTA, Madrid, Spain".

