# Correction: Topological Structure of the Space of Phenotypes: The Case of RNA Neutral Networks

**DOI:** 10.1371/annotation/e1599064-95cc-47c9-bbcc-042202bf0423

**Published:** 2011-12-13

**Authors:** Jacobo Aguirre, Javier M. Buldú, Michael Stich, Susanna C. Manrubia

There was a typographical error in the legend of Figure 7. Different fonts were used.

